# Milk Fat Globule-EGF Factor VIII Attenuates CNS Injury by Promoting Neural Stem Cell Proliferation and Migration after Cerebral Ischemia

**DOI:** 10.1371/journal.pone.0122833

**Published:** 2015-04-13

**Authors:** Cletus Cheyuo, Monowar Aziz, Weng-Lang Yang, Asha Jacob, Mian Zhou, Ping Wang

**Affiliations:** 1 Center for Translational Research, The Feinstein Institute for Medical Research, Manhasset, New York, United States of America; 2 Department of Surgery, Hofstra North Shore-Long Island Jewish School of Medicine, Manhasset, New York, United States of America; Hebei United University, CHINA

## Abstract

The mediators in activating neural stem cells during the regenerative process of neurogenesis following stroke have not been fully identified. Milk fat globule-EGF Factor VIII (MFG-E8), a secreted glycoprotein serves several cellular functions by binding to its receptor, α_v_ β_3_-integrin. However, its role in regulating neural stem cells after stroke has not been determined yet. We therefore, aim to reveal whether MFG-E8 promotes neural stem cell proliferation and migration during stroke. Stroke was induced in wild-type (*Wt*) and MFG-E8-deficinet (*Mfge8^-/-^*) mice by transient middle cerebral artery occlusion (tMCAO). Commercially available recombinant mouse MFG-E8 (rmMFG-E8) was used for mechanistic assays in neural stem cell line, while the in house prepared recombinant human MFG-E8 (rhMFG-E8) was used for *in vivo* administration into rats with tMCAO. The *in vitro* effects of recombinant rmMFG-E8 for the neural stem cell proliferation and migration were determined by BrdU and transwell migration assay, respectively. The expression of cyclin D2, p53 and netrin-1, was analyzed by qPCR. We report that the treatment of rhMFG-E8 significantly improved the neurological deficit score, body weight lost and neural stem cell proliferation in a rat model of tMCAO. Conversely, decreased neural stem cell proliferation was observed in *Mfge8^-/-^* mice in comparison with the *Wt* counterparts underwent tMCAO. rmMFG-E8 stimulated the proliferation of mouse embryonic neural stem cells via upregulation of cyclin D2 and downregulation of p53, which is mediated by α_v_ β_3_-integrin. rmMFG-E8 also promoted mouse embryonic neural stem cell migration via α_v_ β_3_-integrin dependent manner in upregulating netrin-1. Our findings suggest MFG-E8 to promote neural stem cell proliferation and migration, which therefore establishes a promising therapeutic strategy for cerebral ischemia.

## Introduction

Neural stem cells orchestrate embryonic brain development, homeostasis and repair in the adult brain following injury. In the developing brain, pluripotent embryonic neural stem cells proliferate and migrate along various spatial coordinates in response to complex morphogenic gradients, followed by differentiation of the migrated cells to form the adult brain [1, 2]. In the adult brain it has been shown that multipotent neural precursors continue to reside in niches in the subventricular zone and the dentate gyrus, where they contribute to replacement of neurons in the olfactory bulb through the rostral migratory stream [1, 2]. Neural stem cells have also been shown to contribute to brain repair after injury via the secretion of neuroprotective neurotrophic factors as well as tissue regeneration [3, 4]. However, the signaling mechanisms involved in this neuroregenerative process are not fully understood. Deciphering the molecular language of neural stem biology would be critical for the development of novel therapeutics for brain repair and regeneration after stroke.

Milk fat globule-EGF factor VIII (MFG-E8) is a secreted glycoprotein which has two EGF-like domains from the mouse or one EGF-like domain from human at the N-terminal site, and two discoidin domains at the carboxy terminal site from both [5]. The EGF-like domain has an RGD (Arg-Gly-Asp) motif which recognizes integrins α_v_β_3_ and α_v_β_5_. The discoidin domain of MFG-E8 recognizes phosphatidylserine exposed on the cell membranes of apoptotic cells [6, 7]. MFG-E8 is expressed by a wide variety of cells, including immune cells, astrocytes and microglia [8, 9]. Recently, MFG-E8 was demonstrated to protect against acute ischemic brain injury via the suppression of inflammation and apoptosis [10, 11]. However, the effect of MFG-E8 on neural stem cell biology and functional neurological recovery in the setting of cerebral ischemia/reperfusion has not been examined. Here, we report the effects of MFG-E8 on neural stem cell proliferation and migration using a rodent model of cerebral ischemia, as well as *in vitro* approaches.

## Materials and Methods

### Experimental animals

Male Sprague-Dawley rats (300-350g) were purchased from Charles River (Wilmington, MA, USA) and C57BL/6 mice were from Taconic Farms (New York, USA). MFG-E8 knockout (*Mfge8* ^*-/-*^) mice were a kind gift from Dr. Shigekazu Nagata, Kyoto University, Japan. All animals were housed under standard conditions with regular access to standard Purina chow and water. The animal experiments were performed in accordance with the National Institutes of Health guidelines for the use of experimental animals. This protocol was approved by the Institutional Animal Care and Use Committee (IACUC) of the Feinstein Institute for Medical Research.

### Transient middle cerebral artery occlusion (tMCAO)

Animals were fasted overnight except had access to water ad libitum before induction of cerebral ischemia. tMCAO was performed as described previously [10, 12]. Briefly, anesthesia was induced with 3.5% isoflurane in air and subsequently maintained by intravenous boluses of pentobarbital, not exceeding 30 mg/kg. Temperature was maintained between 36.5°C and 37.5°C using a rectal temperature probe and a heating pad connected to a homeothermic monitor (Harvard Apparatus, Holliston, MA). The right femoral artery was cannulated with a PE-50 tubing and connected to Digi-Med blood pressure analyzer, BPA-400 (Micro-Med, Inc. Louisville, KY) for blood pressure monitoring during the tMCAO procedure in rats. The right common carotid artery (CCA) was exposed through a midline neck incision and was carefully dissected free from vagus nerve and fascia, from its bifurcation to the base of the skull. The external carotid artery was dissected and ligated. The internal carotid artery (ICA) was isolated and carefully separated from the adjacent vagus nerve. Microvascular clips were then applied on the common carotid artery and the internal carotid artery. An arteriotomy was then made in the common carotid artery distal to the microvascular clip. A monofilament nylon suture (4–0 for rats and 6–0 for mice) with a rounded tip was then inserted through the arteriotomy into the internal carotid artery and advanced to the middle cerebral artery (MCA) origin to occlude it. A 6–0 silk suture was tied around the intraluminal nylon suture to prevent bleeding. The insertion length of the intraluminal suture required for effective MCA occlusion was 19 mm for rats and 10 mm for mice. The suture was left in place for 90 and 30 min for rats and mice, respectively, after which it was withdrawn to allow reperfusion. Immediately following reperfusion in rats, 200 μl of arterial blood was withdrawn through the cannulated right femoral artery for blood gas analysis. Hemostasis was secured and the cervical wound was then closed in layers and the animals were allowed to recover from anesthesia in a warm and quiet environment. To label proliferating neural stem cells, animals received daily intraperitoneal injections of 5-bromo-2'-deoxyuridine (BrdU), 50 mg/kg, for six days. The first dose of BrdU was given 2 h after inducing cerebral ischemia. The body weight of the animals was measured daily until day 7 post-operation and the animals were sacrificed at day 14. Transcardial perfusion of the brain with ice-cold normal saline followed by 4% paraformaldehyde was performed before removal. To assess the neural stem cells at subventricular zone, coronal brain sections were taken between the levels of +2.7 and -1.3 relative to the bregma in rats, where in mice the sections were taken from +0.38 to -1.42 relative to the bregma [13, 14]. The sections were paraffin-embedded and cut in 6-μm for immunofluorescent and immunohistochemical staining.

### Intracerebroventricular infusion of recombinant human MFG-E8

We have carried-out *in vivo* experiments by utilizing our in-house prepared recombinant human MFG-E8 (rhMFG-E8). On the other hand, for *in vitro* experiments to elucidate the mechanistic pathways we have utilized commercially available recombinant mouse MFG-E8 (rmMFG-E8) purchased from R&D systems, Minneapolis, MN (Catalog No.: 2805-MF-050). According to our previous studies, rhMFG-E8 and rmMFG-E8 obtained from two different species, no noticeable difference in their biological activities between these two protein sources was reported [10, 15, 16]. Continuous intracerebroventricular infusion of rhMFG-E8 or vehicle (Tris buffer, pH 7.5) in rats was achieved by connecting an osmotic pump to a brain infusion kit stereotactically placed in the right lateral ventricle. Briefly, Alzet 2004 mini osmotic pumps, infusion rate 0.25 μl/h, (Durect, Cupertino, CA) were filled either with 0.4 μg/μl rhMFG-E8 or vehicle (Tris buffer) and connected by polyvinylchloride tubing to the brain infusion cannula. The assembly was then primed by immersion in sterile normal saline for 40 h prior to implantation. Immediately after reperfusion following 90-min middle cerebral artery occlusion in rats, the brain infusion cannula was stereotactically placed in the right lateral ventricle at 1.2 mm lateral and 0.8 mm anterior to the bregma. The cannula was inserted to a depth of 4 mm below the pial surface and held in place using loctite 454 instant adhesive. The mini osmotic pump was then buried in a subcutaneous pocket in the midscapular area. Patency of the cannula was assessed when the animal was sacrificed by injecting Evans blue into the polyvinylchloride tubing and watching for outflow through the cannula. If the cannula was found to be blocked, the animal was eliminated from the study and replaced.

### Assessment of neurological function

Neurological deficits in rats were assessed using the Bederson neurological scale [17] which measures global neurological function. Briefly, rats were held by the tail, suspended one meter above the floor and observed for forelimb movement. Normal rats, which extend both forelimbs towards the floor, were scored 0. Rats which displayed forelimb flexion and no other abnormality were scored 1. Rats which showed forelimb flexion in addition to decreased resistance to lateral push towards the paretic side received a score of 2. If rats showed forelimb flexion, decreased resistance to lateral push in addition to circling when allowed to walk on a flat surface, a score of 3 was awarded. Rats were scored at baseline before induction of tMCAO and at 2, 7 and 14 days post-tMCAO.

### Immunofluorescence

Brain tissue sections were de-waxed and rehydrated, followed by microwave antigen retrieval procedure. After blocking non-specific binding using 10% normal goat serum, the following primary antibodies were added and incubated at 4°C overnight: mouse anti-BrdU monoclonal antibody (1:500, Cell Signaling Technology, Danvers, MA), rabbit anti-nestin polyclonal antibody (1:500, Sigma-Aldrich, St. Louis, MO) and rabbit anti-doublecortin polyclonal antibody (1:100, Santa Cruz Biotechnology, Santa Cruz, CA). Next the slides were incubated with respective FITC and TEXAS red-conjugated secondary Abs followed by washing and mounting with DAPI-containing medium (Vectashield H-1200) for fluorescent microscopy.

### Embryonic neural stem cell culture

Embryonic day-14 mouse striata neurospheres were purchased from Stem Cell Technologies (Vancouver, Canada). The neurospheres were cultured and passaged according to the manufacturer’s instructions. In brief, the cryopreserved neurospheres were thawed and cultured in complete embryonic NeuroCult proliferation medium (Stem Cell Technologies) in T-25 flasks and incubated at 37°C in 5% CO_2_ until they reached 100–150 μm in diameter. The neurospheres were then chemically dissociated using the NeuroCult enzymatic dissociation kit according to manufacturer’s instructions. The single cells obtained were then seeded at 5 x 10^6^ cells per T-25 cm^2^ flask containing 10 ml of complete embryonic NeuroCult proliferation medium. The neurospheres were re-passaged when they reached 100–150 μm in diameter. Experiments were performed using passage 3–6 neurospheres. According to the manufacturer, rhEGF at a dose of 20 ng/ml is required for the optimal growth of the embryonic neural stem cells. We therefore arbitrarily maintained rhEGF at trace levels, 1 ng/ml, in the rhMFG-E8-treated and PBS (vehicle) groups.

### qPCR analysis

To assess the effect of rmMFG-E8 on cyclin D2, p53 and netrin-1 gene expression, passage 3–6 neurospheres were chemically dissociated and the cell suspension was divided into 3 aliquots. For the vehicle group, PBS was added to the cell suspension aliquots. In another aliquot, 0.5 μg/ml rmMFG-E8 and 1 μg/ml of purified rat IgG isotype control (Biolegend, San Diego, CA) were added. In the third aliquot, cells were pre-incubated with 1 μg/ml anti-integrin α_V_ antibody (Biolegend, San Diego, CA) for 1 h after which 0.5 μg/ml rmMFG-E8 was added. Each of the aliquots was then plated in triplicate of 2 x 10^6^ cells per well in six-well plates coated with poly-L-ornithine and laminin. The cultures were incubated at 37°C, 5% CO_2_ for 24 h. Total RNA was extracted from cell pellets by TRIzol reagent (Invitrogen, Carlsbad, CA) and reverse-transcribed into cDNA using murine leukemia virus reverse transcriptase (Applied Biosystems, Foster City, CA). PCR reactions was carried out in a 25 μl final volume containing each forward and reverse primer, cDNA and SYBR Green PCR Master mix (Applied Biosystems). The primer sequences are: cyclin D2: forward: CTGGATCCCATCTTGTTGCT, reverse: GCTCCTCTGCCTTCTTTGTG; Netrin-1: forward: CCAAAGGCAAGCTGAAGATG, reverse: TGAGCGATCCACAAACTC; P53: forward: AGTTCATTGGGACCATCCTG, reverse: GTCCATGCAGTGAGGTGATG; β-actin: forward: CGTGAAAAGATGACCCAGATCA, reverse: TGGTACGACCAGAGGCATACAG. The level of mouse β-actin mRNA was used for normalization and the results were expressed as fold change in comparison with control group.

### BrdU assay

Passage 3–4 neurospheres were chemically dissociated using NeuroCult chemical dissociation kit to obtain single embryonic neural stem cells. BrdU at 1 μM was added to each cell suspension and mixed by gentle pipetting. A total of 400 μl of each cell suspension (20,000 cells) were then dispensed into poly-L-ornithine and laminin-coated 8-well chamber slides, in triplicate for each treatment condition. The cultures were incubated at 37°C for 24 h. After fixing at 70% ice-cold ethanol, the cells were then washed and blocked with blocking buffer for 1 h. Next, the cells were incubated with mouse anti-BrdU monoclonal antibody (1:500, Cell Signaling Technology, Danvers, MA) at 4°C overnight. Finally, the slides were incubated with biotinylated goat anti-mouse IgG (BD Pharmingen, San Jose, CA) for 1 h, and then reacted with streptavidin-TEXAS red (Nuclea Biotechnologies, Pittsfield, MA) for 15 min. The cells were mounted with DAPI-containing medium (Vectashield H-1200) for fluorescent microscopy.

### Neurosphere assay

Passage 3 neurospheres were chemically dissociated and the resulting single cell suspension was plated into 48-well culture plates at 4,000 cells per well in triplicate for each experimental group. To assess the effect of rmMFG-E8 on neurosphere formation, 0.5 μg/ml rmMFG-E8 was added to the culture medium consisting of NeuroCult NSC proliferation supplement plus NeuroCult NSC basal medium in a 1:9 ratio plus 1ng/ml rhEGF. An equivalent volume of PBS was added to the culture medium for the vehicle group. The positive control consisted of the medium supplemented with 20ng/ml of rhEGF. The cultures were incubated at 37°C in 5% CO_2_ for seven days. On the seventh day after plating, the neurosphere colonies were counted under light microscopy.

### Neural colony-forming cell assay (NCFCA)

To perform the NCFCA, the following reagents were used; NeuroCult neural colony-forming cell (NCFC) serum-free medium without cytokines, NeuroCult NSC Proliferation Supplement, rhEGF (10 μg/ml), and type 1 bovine collagen solution (3 mg/ml). The medium containing the cells was mixed by gently pipetting and 1.5 ml of this mixture was then dispensed into each of the 35 mm dishes and placed into the incubator at 37°C and 5% CO_2_. The cultures were incubated for 21 days. rmMFG-E8 and PBS were replenished 48 hourly by adding 60 μl of solution to the center of the culture dish. At day 21 of culture, the 35 mm dishes were placed in gridded scoring dishes and the colonies scored as >2 mm, 1–2 mm, 0.5–1 mm or <0.5 mm in diameter under light microscopy.

### Transwell migration assay

The neural stem cell migration was carried-out in Boyden chamber setups with 8 μm-pore membrane, coated with poly-L-ornithine and laminin. A total of 10,000 cells from each of the experimental groups were added to the upper chamber. The lower chamber of the Boyden chamber contained 700 μl of NeuroCult NSC proliferation supplement plus NeuroCult NSC basal medium in a 1:9 ratio, with 500 ng/ml stromal derived factor-1 (R&D systems, Minneapolis, MN) as chemoattractant. The cells were allowed to migrate for 24 h. The transmigrated cells attached to the bottom of the membrane were then fixed in 10% formalin and stained with propidium iodide and counted under light microscopy.

### Statistics

All data are expressed as mean ± SEM and compared by one-way ANOVA and a Student-Newman-Keuls (SNK) test. A Student t-test was used when only two groups were compared. Differences in values were considered significant with p<0.05.

## Results

### MFG-E8 improves neurological functions after cerebral ischemia

**Fig 1:**
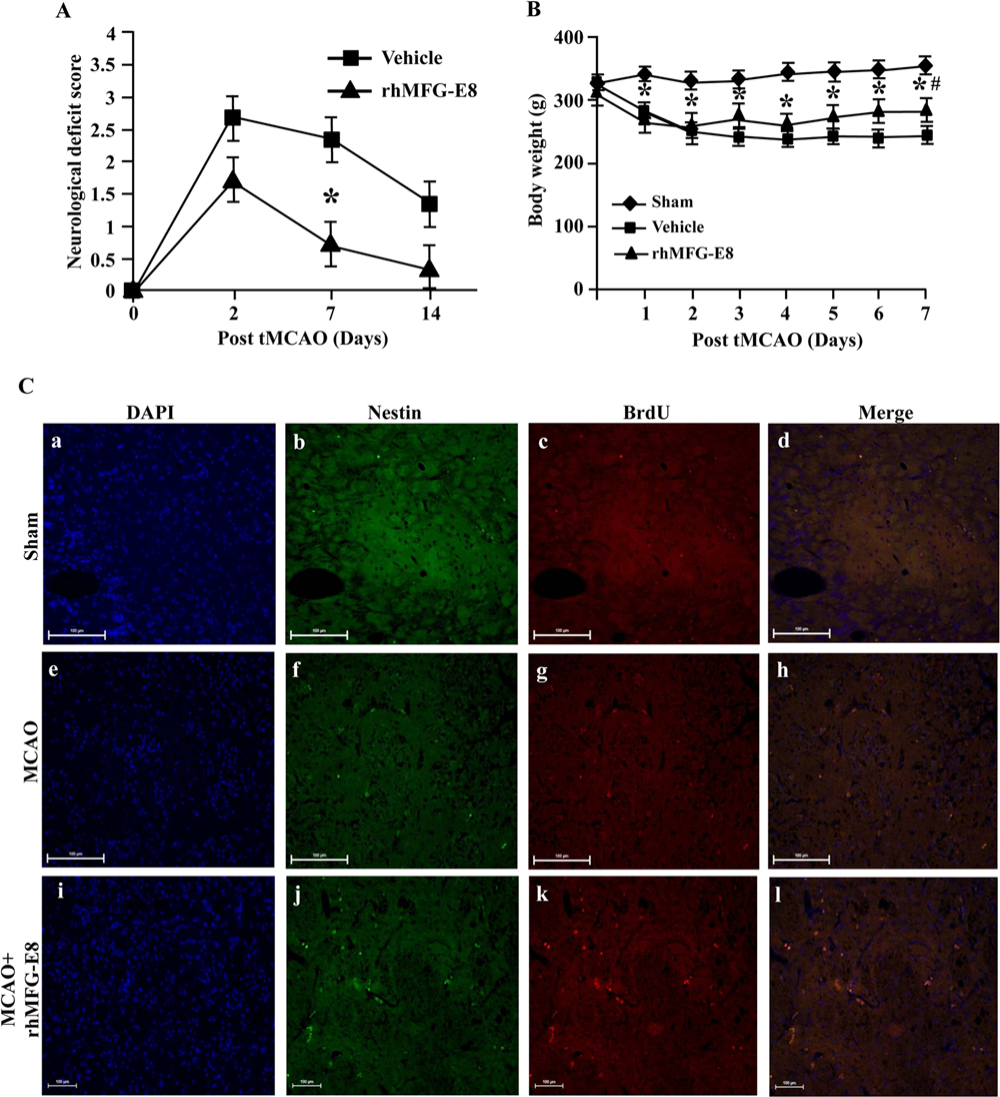
rhMFG-E8 promotes neurological recovery and neural stem cell proliferation after cerebral ischemia. (A) Treatment of rats with rhMFG-E8 significantly decreased neurological deficits at 7 days post-transient middle cerebral artery occlusion (tMCAO) compared with vehicle. Data are presented as mean ± SEM; n = 3 mice/group. The statistical analysis was done by One way Anova and Student-Newman-Keuls (SNK) test. *p<0.05 is considered as statistically significant. (B) Body weight changes of rats within 7 days after tMCAO. Both vehicle and rhMFG-E8-treated groups started to lose weight until day 2 post-tMCAO, at which point the weight of vehicle animals plateaued and rhMFG-E8-treated animals begun to gain weight slowly. Data are presented as mean ± SEM; n = 3 mice/group. The statistical analysis was done by One way Anova and Student-Newman-Keuls (SNK) test. *p<0.05 vs sham; #p<0.05 vs vehicle. (C) The striatum of sham rats (a-d) shows very little baseline neural stem cell proliferative activity as shown by BrdU^+^Nestin^+^ cells. At 7 days post-tMCAO, compared with the vehicle treated animals (e-h), rhMFG-E8 treated rats showed increased number of proliferating neural stem cells (i-l). Scale bar = 100 μm.

A clinically relevant murine model of cerebral ischemia by transient middle cerebral artery occlusion (tMCAO) was used to examine the role of MFG-E8 during stroke. Baseline hemodynamic and arterial blood gas parameters were maintained in our model of tMCAO. No significant changes in the mean arterial blood pressure, pH, bicarbonate, hemoglobin and FO_2_Hb among sham, vehicle and rhMFG-E8-treated animals were noticed (data not shown). Using this model, we found that intracerebroventricular administration of 0.4 μg/μl rhMFG-E8 significantly improved neurological function compared to vehicle at seven days post-tMCAO (Fig 1A). The animals which underwent tMCAO lost body weight compared to sham animals over a seven-day period. However, the tMCAO rats treated with rh showed significant improvement in their body weight at seven days post-tMCAO (Fig 1B).

### MFG-E8 increases neural cell proliferation after cerebral ischemia

**Fig 2:**
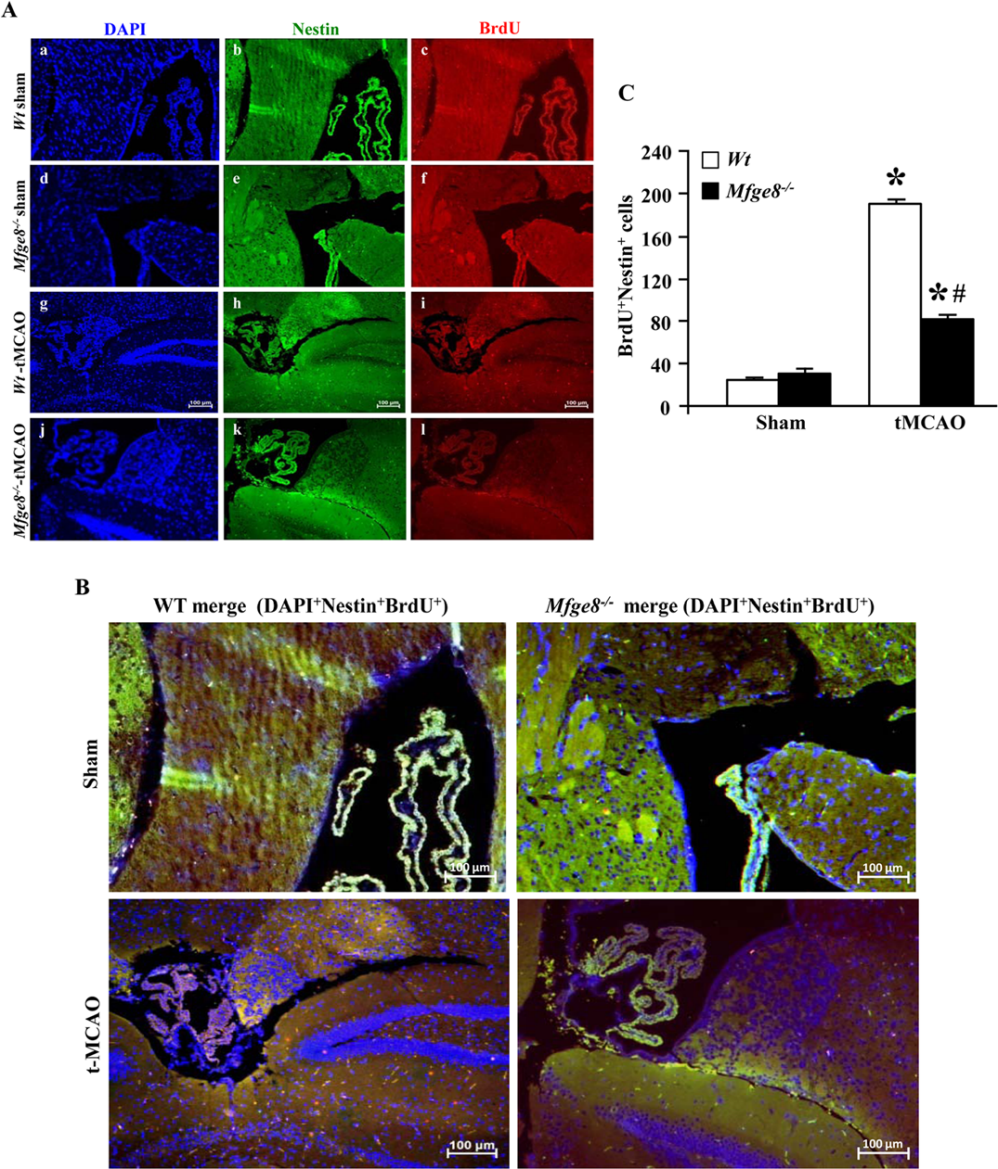
*Mfge8* ^*-/-*^ mice represent decreased neural stem cell proliferation after cerebral ischemia. (A) DAPI, Nestin and BrdU immunofluorescent staining of the subventricular zone in mice. Wild type C57BL/6 (*Wt*) (a-c) and *Mfge8* ^*-/-*^ mice (d-f) showed similar sham baseline neural stem cell proliferation (Nestin^+^ and BrdU^+^ cells). *Wt* mice (g-i) showed increased Nestin^+^ and BrdU^+^ staining compared with the *Mfge8* ^*-/-*^ mice (j-l) at 7 days after tMCAO. Scale bar = 100 μm. (B) Representative merged profiling of DAPI, Nestin and BrdU positive cells showing considerable increase in Nestin+BrdU+ cells in *Wt* tMCAO mice than that of *Mfge8* ^*-/-*^ tMCAO mice. (C) Quantification of the BrdU^+^Nestin^+^ cells showed that *Wt* and *Mfge8* ^*-/-*^ sham mice had similar baseline numbers of proliferating neural stem cells. However, at 7 days post-tMCAO, *Wt* mice showed a 2.3-fold increase in BrdU^+^Nestin^+^ cells compared with *Mfge8* ^*-/-*^ mice. Data are presented as mean ± SEM. *p<0.05 vs WT-Sham; #p<0.05 vs WT-tMCAO; n = 3/group.

Neurogenesis involves neural stem cell proliferation, migration and differentiation, which have been shown to contribute to long-term neurological recovery after cerebral ischemia [18]. To examine the role of MFG-E8 on neurogenesis, after tMCAO, animals were daily injected intraperitoneally with 5-bromo-2'-deoxyuridine (BrdU) for six days and were euthanized on the seventh day. The subventricular zone was then double-stained against nestin, a neural stem cell marker, and BrdU, a marker of proliferation. We observed that the administration of rhMFG-E8 in rats markedly increased the number of proliferating neural stem cells (BrdU+Nestin+) in the striatum at seven days post-tMCAO (Fig 1C). In support, *Wt* mice had significantly higher numbers of proliferating neural stem cells compared to the *Mfge8* ^*-/-*^ mice at seven days post-tMCAO (Fig 2A–2C).

### MFG-E8 promotes neural stem cell proliferation *in vitro*

**Fig 3:**
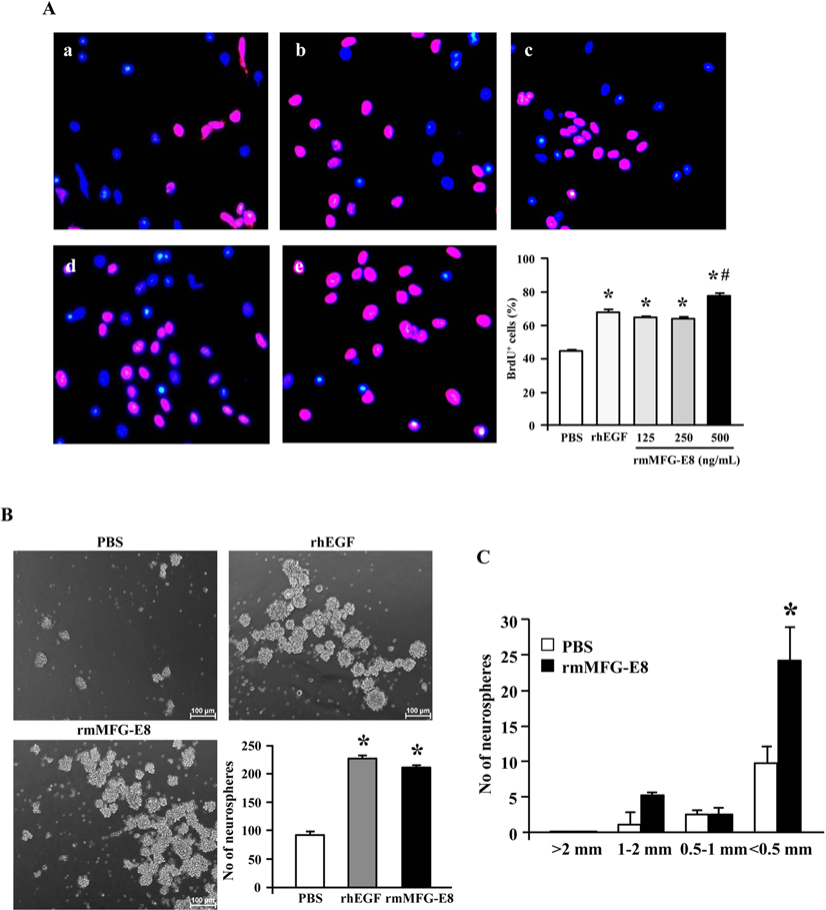
rmMFG-E8 promotes neural stem cell proliferation and neurosphere formation *in vitro*. (A) BrdU (purple) and DAPI (blue) immunofluorescent staining of cultured adherent embryonic neural stem cells after 24 h BrdU exposure and treatment with a. PBS as vehicle, b. 20 ng/ml rhEGF as positive control and increasing doses of rmMFG-E8; c. 125 ng/ml, d. 250 ng/ml and e. 500 ng/ml. Quantification of the number of proliferating adherent embryonic stem cells (BrdU^+^) showed that rmMFG-E8 increased embryonic neural stem cell proliferation in a dose-dependent manner compared with PBS. Data are presented as mean ± SEM. *p<0.05 vs PBS; #p<0.05 vs rhEGF; n = 3/group. (B) rhEGF (20 ng/ml) and rmMFG-E8 (500 ng/ml) increased neurosphere formation compared to PBS. Data are presented as mean ± SEM. *p<0.05 vs PBS; n = 3/group. Scale bar = 100 μm. (C) rmMFG-E8 treatment showed significant increase in the number of neurospheres in the <0.5 mm size categories compared to PBS. *p<0.05 vs PBS; n = 3/group.

We further examined the stimulation of proliferative activity by MFG-E8 in an *in vitro* setting. Mouse embryonic neural stem cells, cultured on poly-L-ornithine/laminin substrate in a medium consisting of neurobasal medium plus proliferative supplement and 1 ng/ml recombinant human epidermal growth factor (rhEGF), were exposed to 1 μM of BrdU for 24 h. Treatment of the cells with rmMFG-E8 dose-dependently increased the BrdU+ fraction, compared to PBS treatment. The optimal dose of 500 ng/ml rmMFG-E8 significantly increased the neural stem cell proliferation in comparison with the positive control of 20 ng/ml rhEGF (Fig 3A). The formation of neurospheres represents the efficiency of neurogenesis [19]. We demonstrated that treatment of embryonic neural stem cell suspension in culture medium with 500 ng/ml rmMFG-E8 resulted in a significantly higher number of neurospheres at day seven in comparison with PBS treatment (Fig 3B). Next, to determine which fraction of neural precursors is affected by MFG-E8, we performed the NeuroCult neural colony-forming cell assay (NCFCA). The NCFCA is a 21-day collagen based semi-solid assay which categorizes neural colonies into sizes >2 mm, 1–2 mm, 0.5–1 mm and <0.5 mm based on the rate at which constituent cells form secondary neurospheres. NCFCA colonies >2 mm, 1–2 mm, 1–0.5 mm and <0.5 mm in size generate secondary neurospheres at 100%, 80%, 53% and 28% rate respectively. Tertiary neurospheres are formed 100% and 50% of the time by >2 mm and 1–2 mm sized colonies. Thus, cells in colonies >2 mm in diameter at day 21 of culture are considered bona fide neural stem cells and colonies 1–2 mm are considered rich in neural stem cells. Colonies 0.5–1 mm and <0.5 mm are considered to be mostly neural progenitor cells [19]. We found that the addition of rmMFG-E8, 500 ng/ml, 48 hourly to the NCFCA tended to increase the 1–2 mm colony fraction compared to PBS. In addition, rmMFG-E8 also had a tendency to increase the <0.5 mm fraction compared to PBS (Fig 3C). Collectively, these data indicate that the treatment of rmMFG-E8 led to generate neural progenitor cells which could be beneficial in neurogenesis after stroke.

### rmMFG-E8 upregulates cyclin D2 but downregulates p53 expression via α_v_β_3_-integrin in neural stem cells

**Fig 4:**
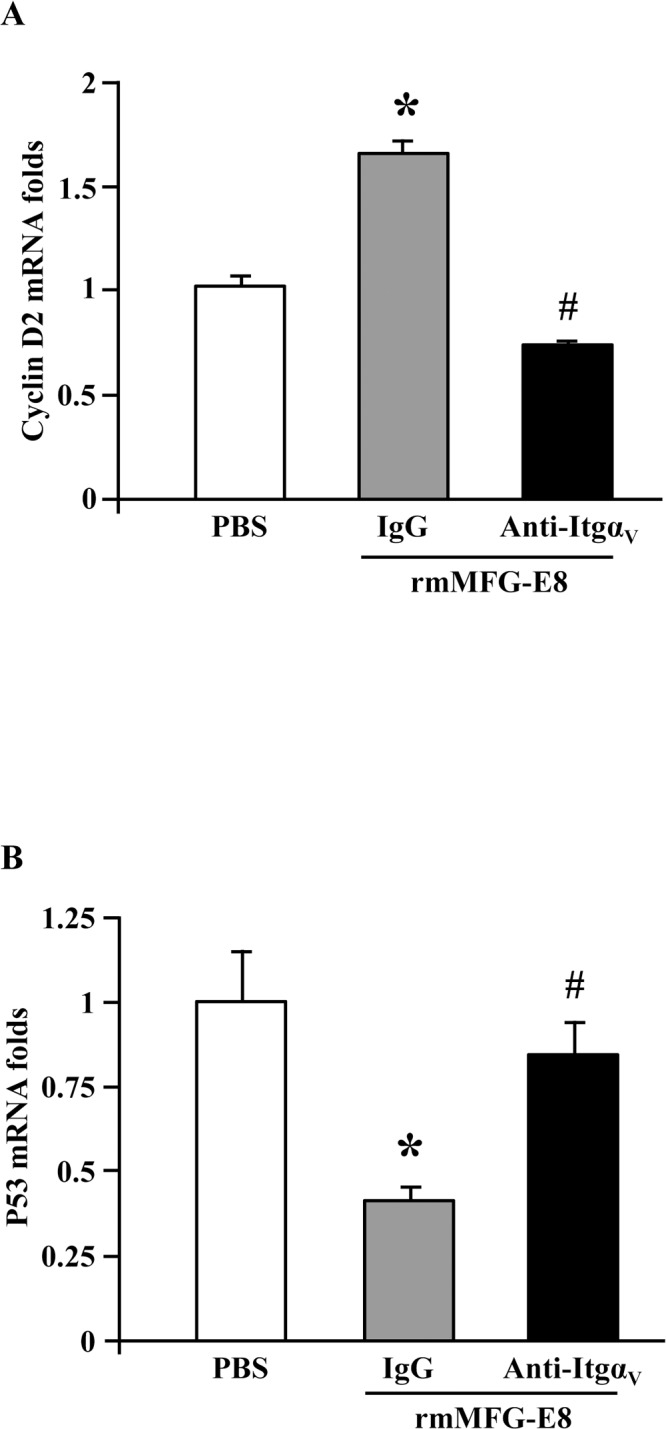
rmMFG-E8 upregulates cyclin D2 and downregulates p53 expression in neural stem cells. (A) rmMFG-E8 upregulates cyclin D2 via integrin α_V_ (Itgav). Treatment of cultured mouse embryonic neural stem cells with 500 ng/ml rmMFG-E8 significantly increased cyclin D2 gene expression compared with PBS. Pre-treatment of the mouse embryonic neural stem cells with 1 mg/ml anti-Itgav antibody abrogated the upregulation of cyclin D2 expression by rmMFG-E8, but not 1 mg/ml control IgG. Data are presented as mean ± SEM. *p<0.05 vs PBS; #p<0.05 vs IgG; n = 3/group. (B) rmMFG-E8 downregulates p53 via integrin α_V_. Treatment of cultured mouse embryonic neural stem cells with 500 ng/ml rmMFG-E8 significantly decreased p53 gene expression compared with PBS treatment. Pre-treatment of the mouse embryonic neural stem cells with 1 mg/ml anti-Itgαv antibody prevented the downregulation of p53 by rmMFG-E8, but not 1 mg/ml control IgG. Data are presented as mean ± SEM. *p<0.05 vs PBS; #p<0.05 vs IgG; n = 3/group.

Since cyclin D2, a highly conserved protein which controls G1 to S transition in the cell cycle, has been reported to play an essential role in neural stem cell proliferation [20, 21], we aimed to analyze cyclin D2 expression in rmMFG-E8-treated neural stem cells. We demonstrated that rmMFG-E8 treatment significantly upregulated the cyclin D2 mRNA levels in cultured embryonic neural stem cells in comparison with PBS. Blocking the MFG-E8 receptor with anti-integrin α_V_ antibody prevented the upregulation of cyclin D2 by rmMFG-E8 (Fig 4A). To further investigate the neural stem cell proliferative activity of MFG-E8, we examined its effect on the tumor suppressor gene, p53, which regulates stem cell division by modulating the cell cycle [22]. We observed that rmMFG-E8 treatment of cultured embryonic neural stem cells dramatically reduced p53 mRNA levels in comparison with PBS treatment. Blockage of the MFG-E8 receptor using anti-integrin α_V_ antibody resulted in the restoration of p53 mRNA levels back to those of the PBS-treated group (Fig 4B). Thus, our results have shown that MFG-E8 promotes neural stem cell proliferation by upregulating cyclin D2, while suppressing p53 expression through α_v_β_3_-integrin.

### MFG-E8 enhances neural stem cell migration via α_v_β_3_-integrin-mediated upregulation of netrin-1

**Fig 5:**
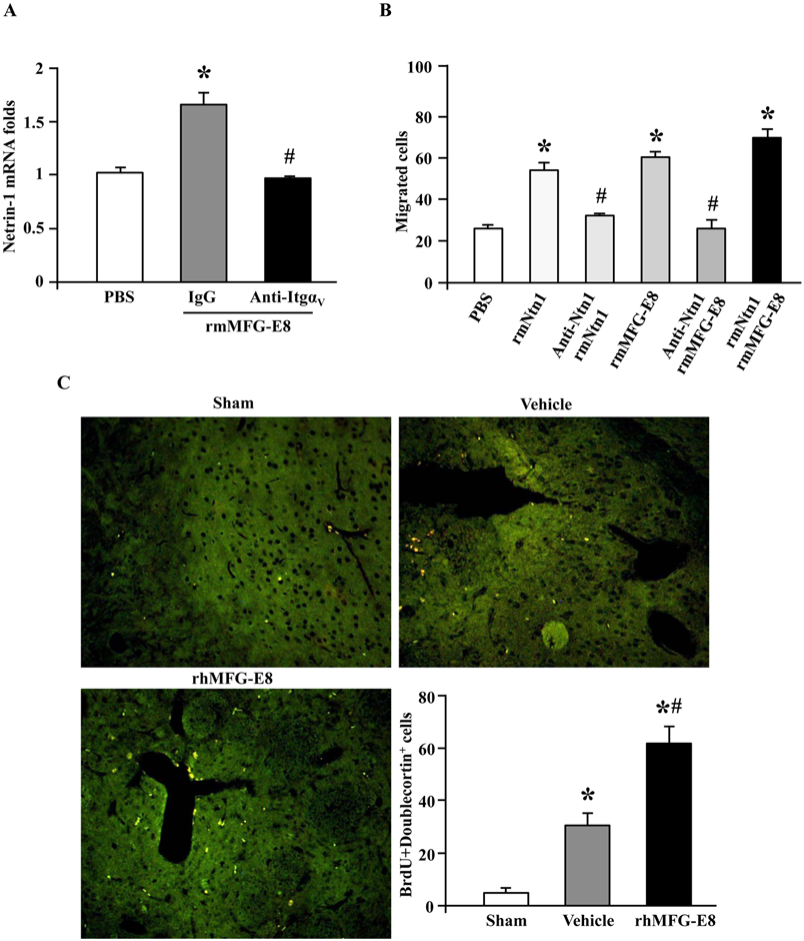
rmMFG-E8 promotes neural stem cell migration via netrin-1. (A) rmMFG-E8 upregulates netrin-1 via integrin α_V_. Pre-treatment of mouse embryonic neural stem cells with 1 mg/ml anti-Itgav antibody prevented rmMFG-E8 mediated upregulation of netrin-1, but not 1 mg/ml control IgG. Data are presented as mean ± SEM. *p<0.05 vs PBS; #p<0.05 vs IgG; n = 3/group. (B) Quantification of the mouse embryonic neural stem cell migration through the transwell showed that rmMFG-E8 promotes embryonic neural stem cell migration through netrin-1. Both rmNtn-1 and rmMFG-E8 increased cell migration compared with PBS. Pre-treatment of the mouse embryonic neural stem cells with anti-Ntn-1 antibody blocked the effects of both rmNtn-1 and rmMFG-E8 on cell migration. Treatment of the mouse embryonic neural stem cells with a combination of rmNtn-1 and rmMFG-E8 had an additive effect in increasing cell migration. Data are presented as mean ± SEM. *p<0.05 vs PBS; #p<0.05 vs rmNtrn1/rmMFG-E8; n = 3. (C) The effect of rhMFG-E8 on neural precursor migration in rats. Doublecortin and BrdU double immunofluorescent staining of the striatum was performed to identify migrating neuroblast (Doublecortin^+^BrdU^+^) at 7 days after tMCAO. Representative images are shown at the top of the figure. tMCAO increased the number of Doublecortin^+^BrdU^+^ cells in the striatum compared to sham baseline. rhMFG-E8 treatment significantly increased the number of Doublecortin^+^BrdU^+^ cells compared with the vehicle group. Data are presented as mean ± SEM. *p<0.05 vs sham; #p<0.05 vs vehicle; n = 3/group.

Netrin-1 (Ntn-1) is a secreted axonal guidance molecule which has been shown to promote migration of neural stem cells [23]. We first examined the effect of MFG-E8 on Ntn-1 expression. The mRNA levels of Ntn-1 in embryonic neural stem cells were increased by rmMFG-E8 treatment, while they were inhibited by incubation of anti-integrin α_V_- antibody (Fig 5A). We further examined whether the upregulation of Ntn-1 expression by rmMFG-E8 affected neural stem cell migration. The treatment of the plated embryonic neural stem cells with recombinant Ntn-1 significantly increased cell migration in comparison with PBS treatment (Fig 5B). Pretreatment with an anti-Ntn-1 neutralizing antibody prevented the Ntn-1-mediated increase in cell migration (Fig 5B). Similarly, pretreatment with the anti-Ntn-1 neutralizing antibody also blocked rmMFG-E8-mediated increase in neural stem cell migration (Fig 5B). Finally, we showed that treatment of the neural stem cells with a combination of Ntn-1 and rmMFG-E8 had an additive effect on cell migration (Fig 5B). MFG-E8-mediated stimulation of neural stem cell migration was also demonstrated in rats underwent tMCAO, where their striatum were stained against BrdU and doublecortin, a marker of migrating neuroblasts (Fig 5C). rmMFG-E8-treated animals had higher number of BrdU^+^doublecortin^+^ cells than the vehicle-treated ones (Fig 5C).

## Discussion

There are emerging therapies in experimental stroke which aim to amplify neurogenesis by modifying the microenvironment of neural stem cell niches in order to promote proliferation, migration, differentiation and integration of neural precursors within the ischemic brain tissue. The mediators controlling neural stem cell proliferation, migration and their differentiation into neurons have several therapeutic applications for brain injury. Experimental stimulation of endogenous neural stem cells has been demonstrated to promote neuronal regeneration. Infusion of a variety of neurotrophic growth factors, including basic fibroblast growth factor, epidermal growth factor and brain-derived neurotrophic factor, into the lateral ventricle of the rodent with stroke increases neurogenesis [24–26]. Transplantation of exogenous neural stem cells, with or without modification with biologic agents, has also been shown to have therapeutic potential for various brain diseases [27]. Treatment of stroke in the rodent with bone marrow mesenchymal cells (MSCs) after stroke stimulates brain parenchymal cells to secrete an array of neurotrophic factors, leading to augmentation of neurogenesis [28].

Recently, MFG-E8, an endogenously secreted glycoprotein, has been shown to have an essential role in promoting the regeneration of injured intestinal mucosa by accelerating cellular migration and proliferation via protein kinase C-dependent pathway [29]. In agreement with these findings, we have previously demonstrated an enhanced cellular regenerative potential of rhMFG-E8 in treating rats with radiation-induced intestinal injury [30]. Other than the intestinal tissues, MFG-E8 also promoted neovascularization after surgically induced hindlimb ischemia via induction of the vascular endothelial growth factor and AKT-dependent pathways [31]. In the current study, we have demonstrated a novel mechanism through which MFG-E8 modulates the proliferation and migration of both embryonic neural stem cells *in vitro* and adult neural stem cells *in vivo* during cerebral ischemia. The elucidation of this novel pathway involving integrin α_v_-mediated regulation of cyclin D2, p53 and netrin-1 will provide an innovative therapeutic tool for increasing the proliferative and migratory potential of neural stem cells during stroke.

Following stroke, animals subsequently exhibit a variety of neurological deficits. In the current study we have utilized one of the most commonly used strategies to evaluate neurological deficits after cerebral ischemia. The Bederson scale is a global neurological assessment that was developed to measure neurological impairments following stroke [17]. The Bederson scale is based upon three major tests which include: i) forelimb flexion, ii) resistance to lateral push and iii) circling behavior. A grading scale of 0–3 is used to assess behavioral deficits after stroke. In accordance with our findings, others also have adopted this scale to validate their ischemic animals with higher score indicating the severity of neurological deficits than the non-ischemic animals [32]. Nevertheless, the neurological ratings on this scale may be limited because of their subjective nature, even though it is easy to perform. In addition, the deficits on the Bederson scale resolve quickly in many common stroke models, rendering it less useful for the detection of long-term deficits after stroke. In our study, we performed the cerebral ischemia in animals by transiently blocking the middle cerebral artery and evaluated the short term neurologial defects rather than the long-term deficits by Bederson scale, which therefore ruled-out any limitation of using this scale in our system. Apart from that, to minimize the experimental error we have also performed our studies in an unbiased double blinded fashion.

The redundant expression of the cell cycle regulatory proteins, cyclins, in most cells implies that the modulation of a specific cyclin may have little effect on cell proliferation. However, in cells in which one particular cyclin is predominantly expressed, alterations in the level of expression have profound effects. Kowalczyk *et al*. demonstrated that in mouse neural precursors, Cyclin D2 is the only D-type cyclin expressed, thus making it essential for neural stem cell proliferation [20]. Cyclin D2 knockout mice have been shown to have suppressed neurogenesis, which results in a phenotype characterized by learning deficits [33]. Pharmacological suppression of cyclin D2 expression has also been shown to decrease the proliferation of neural stem cells [34]. In our study, we have shown that the treatment of rmMFG-E8 to the cultured mouse embryonic neural stem cells significantly upregulated the cyclin D2 expression and that the rmMFG-E8-mediated cyclin D2 upregulation occurred through its receptor, α_V_β_3_-integrin (Fig 4). The tumor suppressor gene, p53 has been reported to modulate neural stem cell proliferation [22]. Our study revealed that the *in vitro* treatment of neural stem cells with MFG-E8 downregulated p53 gene expression via α_V_β_3_-integrin signaling, implying with the fact that rmMFG-E8-mediated neural stem cell proliferation occurred through the downregulation of p53 expression. In accordance with our findings, using the p53 knockout mice Liu *et al*. have demonstrated that the p53 knockout resulted in increased neural stem cell proliferation and formation of neurospheres [35].

The migration of neural stem cells from the niche microenvironment to sites of brain injury is a critical step in neurogenesis. We have shown that MFG-E8 promotes the migration of neural stem cells via integrin α_V_-mediated upregulation of netrin-1. Netrin-1 is an axonal guidance molecule which promotes neuronal migration either through Deleted in colorectal cancer (DCC)-mediated chemoattraction or by chemorepulsion mediated by Unc5 proteins, alone or with DCC [36]. We demonstrated that MFG-E8-mediated integrin α_V_-signaling upregulated netrin-1 expression. The addition of recombinant netrin-1to neural stem cells in the upper chamber of a transwell promoted their migration, suggesting a mechanism of chemorepulsion. The addition of MFG-E8 to the neural stem cells in the upper chamber also resulted in increased migration. Neutralization of netrin-1 using monoclonal antibodies however, prevented MFG-E8-mediated neural stem cell migration, indicating that MFG-E8 upregulates netrin-1 secretion, leading to neural stem cell migration by chemorepulsion.

The recombinant MFG-E8 used in this study was prepared from mouse or human cDNAs. The nascent mouse or human MFG-E8 mRNA contains an N-terminal signal peptide sequence followed by EGF-like domains and C-terminal discoidin domains [5]. In mouse, the signal peptide sequence belongs to 1–23 amino acids (aa), and in human it is 1–24 aa. While preparing the mouse or human recombinant MFG-E8 protein, the commercial vendor, R&D systems cloned the cDNAs correspond to the mature peptide belonging to 23–463 aa for mouse and 24–387 aa for human, respectively lacking their signal peptide sequence. According to the NCBI protein homology database, we have noticed 68% homology between mouse and human MFG-E8 in their amino acid sequences, which therefore may exhibit similar biological functions when injected into mouse or rats during acute inflammatory disease conditions. In our previous studies, rats pre-treated with recombinant human MFG-E8 exerted dose dependent benefits in sepsis [15]. Hence, we carried-out our *in vivo* studies utilizing recombinant human MFG-E8. On the other hand, based on our previous reports for elucidating the mechanism of MFG-E8, herein we utilized recombinant murine MFG-E8 for our mechanistic approaches using murine cells [16, 37].

Unlike our finding, recently Neher *et al*. reported reduced brain atrophy in MFG-E8 deficient animals after focal cerebral ischemia [38], which we think could be due to the differences in animal models used in independent studies. The animal model used in our previous study [10] and in current study is considerably different from the one used by Neher *et al* [38]. In our current study, we induced cerebral ischemia by mechanically blocking a major blood vessel in the brain, similar to what happens in human ischemic stroke. On the other hand, Neher *et al*. induced stroke chemically, using endothelin-1. Even though, there is cerebral ischemia in both situations, the intrinsic biological activity of endothelin-1 cannot be ignored. In addition to causing vasoconstriction, leading to cerebral ischemia, endothelin-1 has also been shown to regulate the expression of several proteins which in turn may generate different findings. For example, endothelin-1 is known to increase the expression of osteopontin, a pro-inflammatory protein [39], which strongly competes with MFG-E8 for binding to the integrin α_v_β_3_ receptor [40], thereby opposing MFG-E8 function. It is clear from the above that we, together with other investigators, have firmly established the neuroprotective role of MFG-E8 in a clinically relevant animal model of ischemic stroke. The current study is going a step further to delineate the novel effects of MFG-E8 on neurogenesis induced by cerebral ischemia and the molecular mechanisms involved thereof. In the present study we have provided novel findings on the effects of recombinant MFG-E8 for neuronal stem cell migration *in vivo* as well as *in vitro* by identifying previously unexplored upstream (α_v_β_3_-integrin) as well as downstream (Cyclin D2, P53 and Netrin-1) pathways. Even though the lack of inclusion of infarct size data is a limitation of our study which might provide some additional confirmation, we strongly believe that our supportive *in vivo* as well as *in vitro* stem cell migration and proliferation data reasonably draw a convincing conclusion of our original hypothesis.

Taken together, we have defined a novel mechanism by which MFG-E8 promotes neural stem cell proliferation and migration via integrin α_V_-mediated modulation of cyclin D2, p53 and netrin-1 expression. Currently, there is no known therapy for promoting neural regeneration following ischemic stroke. We have previously shown that MFG-E8 decreases acute ischemic brain injury by mitigating inflammation and apoptosis. The current study has answered the critical question of whether an extension of MFG-E8 treatment beyond the acute stages of ischemic stroke would promote recovery. The data in this study has shown that MFG-E8 treatment would promote neurological recovery by increasing neurogenesis. Thus, MFG-E8 has the potential to be further developed as a therapeutic for acute ischemic stroke and also an adjunct for promoting neuroregeneration during rehabilitation.
